# The State of Peptide Detectability in Computational Proteomics and Guidelines for AI Applications

**DOI:** 10.34133/csbj.0037

**Published:** 2026-04-07

**Authors:** Vincent Schilling, Aleksandar Anžel, Joerg Doellinger, Peter Lasch, Dominik Heider, Georges Hattab

**Affiliations:** ^1^ Robert-Koch Institute, Center for Artificial Intelligence in Public Health Research (ZKI-PH), Berlin 13353, Germany.; ^2^ Department of Mathematics and Computer Science, Freie Universität Berlin, Berlin 14195, Germany.; ^3^ Robert-Koch Institute, Center for Biological Threats and Special Pathogens: Proteomics and Spectroscopy (ZBS 6), Berlin 13353, Germany.; ^4^ Institute of Medical Informatics, University of Münster, Münster 48149, Germany.

## Abstract

Artificial intelligence (AI) techniques have transformed proteomics and computational biology over the past decade, particularly in mass spectrometry tasks such as fragment spectra prediction, retention time prediction, and peptide detectability prediction. However, as the volume of proteomics data grows, the need for robust, reproducible, and comparable AI applications has become increasingly urgent. Here, we present a comprehensive scoping review of AI techniques for peptide detectability, an essential and rapidly evolving problem in computational proteomics. By analyzing more than 25 peer-reviewed methods published between 2006 and 2025, we identify a persistent discrepancy between increasing algorithmic sophistication and consistent adherence to established machine learning standards. Current studies frequently exhibit heterogeneity in dataset construction, limited transparency in model design and evaluation, and restricted reproducibility. To address these challenges, we propose actionable guidelines focused on transparent technical reporting, rigorous dataset separation, comprehensive performance evaluation, and reproducibility. Encouragingly, recent tools demonstrate that such standards can be met in practice. Furthermore, we outline a future research agenda that emphasizes the integration of structural biology frameworks, the transition toward data-independent acquisition datasets, and the development of explainable AI to ensure models are both biologically interpretable and transferable across evolving instrument platforms. While centered on peptide detectability, these principles offer valuable insights and can inform future work across a wide range of computational proteomics applications.

## Graphical Abstract

**Figure:**
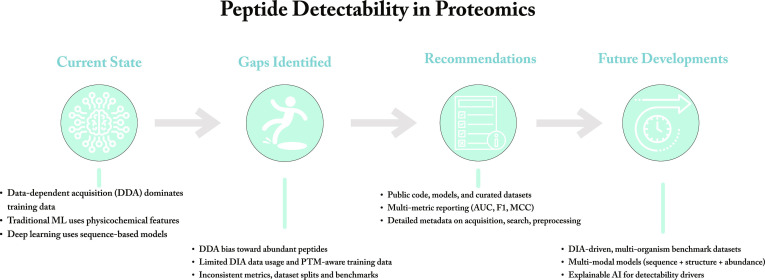

## Introduction

Mass spectrometry (MS) is a widely used technique in high-throughput proteomics for the identification and quantification of proteins and peptides [1]. The coupling of liquid chromatography (LC) to MS serves as a preliminary separation step to reduce sample complexity. The term “resolution” is used to describe the ability to separate 2 or more compounds. High resolution allows for more effective separation of peptides, thereby increasing the detectability of the analytes, or compounds whose chemical constituents are being identified and measured. A standard bottom-up liquid chromatography–tandem mass spectrometry (LC–MS/MS) workflow consists of 5 distinct steps. First, proteins are extracted from organisms by cell lysis and denaturation. Second, the proteins are digested into peptides using an enzyme that cleaves the protein at specific amino acids (AAs). In most cases, trypsin is used due to its high degree of cleavage specificity, which results in the generation of peptides within the optimal mass range [2,3]. Third, the resulting peptides are separated by LC. Fourth, the peptides are introduced into the mass spectrometer, where they are first measured as intact precursor ions (MS1). In proteomics, a precursor is the initial ionized peptide or protein fragment that is analyzed by mass spectrometry for identification. Selected precursor ions are then fragmented in the gas phase, typically by collision-induced dissociation (CID) or higher-energy collisional dissociation (HCD), producing fragment ions that are analyzed in a second stage of mass spectrometry (MS2). Fifth, the resulting MS/MS spectra are interpreted to identify peptides and infer the proteins from which they originate.

**Fig. 1.**
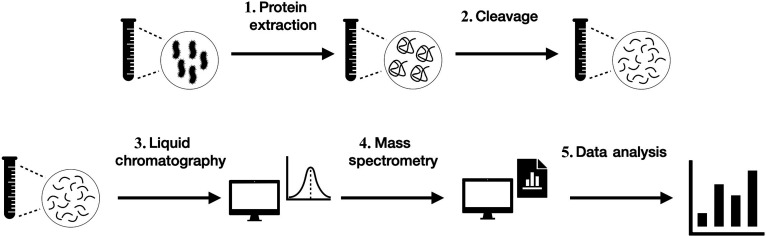
The standard 5-step workflow for a liquid chromatography–tandem mass spectrometry (LC–MS/MS) experiment using bacteria as the organism. It consists of 5 steps: (1) Proteins are extracted by cell lysis and denatured. (2) Proteins are cleaved or digested into smaller peptides. (3) Peptides are separated by liquid chromatography based on their physicochemical properties. (4) Peptides enter the mass spectrometer, are measured (MS1), fragmented, and fragment ions are analyzed (MS2). (5) The resulting mass spectra are further analyzed.

In this process, peptides are separated based on their mass-to-charge (*m*/*z*) ratio in an electric field, and the resulting data are further analyzed [4]. The entire workflow, as shown in Fig. 1, forms the basis of MS-based proteomics, which includes both targeted and untargeted approaches. Targeted mass spectrometry approaches include selected reaction monitoring (SRM), multiple reaction monitoring (MRM), and parallel reaction monitoring (PRM). They are used for the analysis, monitoring, and identification of specific predefined metabolites or proteins of interest [5–7]. Untargeted approaches include data-dependent acquisition (DDA) and data-independent acquisition (DIA). The main difference is in the selection of ions for fragmentation. DDA isolates individual peptide sequences in narrow windows, while DIA uses a series of *m*/*z* windows to analyze all peptides within the sample. While the application of the 5-step workflow provides a systematic framework for peptide identification, the resulting data typically reveal only a subset of the total expected peptide sequences for any given protein. The reason for the discrepancy in peptide identification across multiple experiments, where some peptides are consistently identified while others remain unidentified, is not fully understood. The majority of researchers agree that the inherent physicochemical properties of the peptide, including retention time, co-elution, ionizability, and abundance, influence the probability of identification [8–14]. In fact, by exploiting these properties, several research groups have used traditional machine learning (ML) and deep learning (DL) models to predict peptide detectability.

In recent years, a wide variety of fields have embraced the use of artificial intelligence (AI) techniques to harness the ever-expanding and increasingly intricate datasets. A similar trend can be observed in the field of proteomics, with the first examples dating back to 2006 [15,16]. A variety of AI techniques have been developed for the various stages of the LC–MS/MS process. Researchers have used AI techniques specifically designed for predicting digestibility [17–21], retention time [22–26], and fragment spectra [27–32]. The first two are often used as predictors of peptide detectability, which is the primary goal of this research. To the best of our knowledge, there are only 2 reviews that provide a comprehensive overview of the application of AI in the field of computational proteomics with the focus on mass spectrometry [15,16], with only one focusing exclusively on DL techniques [16], and none offering a dedicated review of peptide detection methods developed over the past 25 years (since 2000).

This article compares the performance, techniques, data, usability, and reproducibility of AI techniques for predicting peptide detectability. It is also shown that a substantial number of published AI techniques in peptide detectability fail to meet established ML standards. These include data preprocessing, usability, data quality improvement, feature selection and engineering, model selection, hyperparameter tuning, appropriate evaluation metrics, model interpretability, reproducibility, and comparability between techniques. Guidelines for applying these standards are provided and the performance and applicability of the tools in peptide detectability in quantitative MS-based proteomics are discussed.

## Results

To systematically evaluate the current state of ML in peptide detectability, this section follows the successive stages of the proteomics pipeline across 5 distinct parts. We first detail the differences in mass spectrometry data acquisition techniques and their critical impact on downstream ML applications. Next, we review the broader applications of ML and DL in MS-based proteomics before focusing specifically on the fundamental principles and physicochemical characteristics that influence peptide detectability. The fourth part provides a comprehensive overview of existing detectability tools, detailing their underlying ML/DL architectures and input features. Finally, we present an in-depth analysis of selected tools to illustrate our primary finding: current ML standards for peptide detectability remain highly fragmented and heterogeneous. Specifically, we highlight pervasive issues within the field, including the scattered reporting of performance metrics, unclear dataset splits (training, testing, and validation), biased comparisons, and a widespread lack of transparent access to underlying data and source code.

### MS-based proteomics: Acquisition types

MS-based proteomics provides a powerful framework for profiling biological systems, enabling researchers to describe biochemical states by estimating the relative abundance of proteins or, through the use of reference standards, measuring absolute protein concentrations. These strategies are generally categorized into targeted quantitative methods and untargeted discovery-based approaches.

As mentioned in the Introduction, targeted mass spectrometry techniques such as SRM, MRM, and PRM are designed for the precise analysis, monitoring, and quantification of specific, predefined metabolites or proteins [5–7]. These techniques offer high sensitivity and reproducibility by focusing on selected targets rather than scanning the entire proteome. The advantages of this approach include high sequence-based selectivity, high sensitivity, rapid cycle execution, and high reproducibility. The disadvantages of this approach include the need to develop the technique prior to acquisition, which involves the selection of precursor ions and optimization of MS parameters. In proteomics, a precursor is the initial ionized peptide or protein fragment that is analyzed by mass spectrometry for identification. In our analysis, only one study used targeted mass spectrometry data, and only as a component of their overall data [33]. Because targeted approaches monitor a restricted list of preselected peptides rather than the full range of observable sequences, they can yield lower sequence diversity than DDA or DIA techniques. While a targeted method can achieve high depth for its specific targets by providing a comprehensive list, the lack of broad peptide coverage makes it less suitable for training generalizable ML models, which require large datasets with low bias to learn the underlying features of peptide detectability. Furthermore, since targeted MS requires prior knowledge of which peptides are detectable to build the inclusion list, it is inherently biased toward proteotypic peptides. This makes it an impractical data source for studies attempting to assess or predict the general detectability of peptides.

Untargeted approaches include the DDA and DIA techniques. The main difference is in the selection of ions for fragmentation. DDA isolates individual peptide sequences in narrow windows, yielding easily interpretable spectra, but is limited by MS acquisition speed, which limits peptide coverage and reduces reproducibility due to undersampling and stochastic precursor sampling. When precursors have similar *m*/*z* ratios, spectrum interpretation becomes challenging [34]. The majority of available datasets used by our selected tools are still based on this technique. Other acquisition types may be more suitable as a basis for ML techniques. DIA, on the other hand, uses a defined *m*/*z* window to analyze all peptides in the sample, allowing for unbiased peptide quantification and greater proteome coverage [35]. While DIA provides comparable accuracy, reproducibility, and consistency to SRM, it generates complex spectral data that require advanced computational analysis [36]. DDA selects abundant ions for fragmentation, while DIA fragments all ions within a given *m*/*z* range, improving coverage. Both techniques use LC–MS/MS for peptide detection, with DIA becomes more prevalent due to its reproducibility, improved coverage, and improved spectral analysis algorithms [16].

Only the tool PREGO of Searle et al. [33] from the ones we reviewed incorporated DIA data in their training data, while the majority relied on DDA-based datasets. The predominance of tools trained on DDA data is not merely a result of its longer-standing availability and wider adoption in early proteomics. A more fundamental barrier is the analytical complexity inherent to DIA data. In DDA, the mass spectrometer isolates a single precursor for fragmentation, providing a 1:1 mapping between a peptide sequence and its resulting MS2 spectrum. In contrast, DIA utilizes wide isolation windows that fragment multiple precursors simultaneously. This results in highly convolved spectra where fragment ions from different peptides are interleaved. Using such data for training ML models requires solving nontrivial deconvolution and attribution problems to ensure that the features being learned are correctly mapped to the right peptide, which have only in recent history been tackled. This problem explains why the field has historically relied on the cleaner, more straightforward training sets provided by DDA workflows. We did not observe performance differences that could be directly attributed to acquisition type, partly because no classifiers were trained exclusively on SRM or DIA data. It is important to note that DDA introduces inherent biases. Specifically, its stochastic precursor selection tends to favor more abundant peptides, potentially skewing the training data and limiting the diversity of observed peptides. In contrast, DIA offers a more comprehensive and systematic sampling of the proteome, which may provide a less biased foundation for developing peptide detectability models. This discrepancy between the increasing adoption of DIA in modern proteomics and the predominance of DDA-derived training data highlights a potential limitation in the representativeness of current ML models.

All 3 acquisition strategies (SRM, DDA, and DIA) generally produce data in similar formats, which is inherently dependent on the mass spectrometer used. In the context of ML, the main differences lie in the volume of data generated, the *m*/*z* window scanned, the reproducibility of runs for data consistency, and the reproducibility of peptide identification across runs. The latter is essential to ensure data consistency and is critical to the successful development of ML algorithms for predicting peptide detectability.

### ML and DL applications in proteomics

In this section, we aim to provide an overview of ML and DL applications in computational proteomics. ML defines the ability of an algorithm to learn from data to solve a problem by applying mathematics and identifying meaningful patterns [37,38]. Over the past 20 years, as computing power has increased, ML has become more prevalent in applications and research, outperforming traditional algorithms. In the last decade, the amount and availability of data have increased dramatically. The already existing shallow artificial neural networks became more complex to accommodate this, introducing the term DL for it.

One of the fields where traditional ML and DL have been widely applied is MS-based proteomics. The data generated by mass spectrometers can be very large and complex and therefore difficult to analyze using traditional statistical techniques. When trying to identify peptide sequences from spectral data or predict MS spectra, traditional ML and DL approaches have been shown to be very good at solving these tasks [39,40]. A common task in computational proteomics is the prediction of peptide digestibility. In MS experiments, proteins are digested into smaller peptides using enzymes such as trypsin. Missed cleavages can occur, and accurately predicting these missed cleavages can improve protein and peptide identification, peptide detectability, and protein abundance estimation. Several techniques using ML and DL techniques have been published on this topic [17–21]. Another area where traditional ML and DL have been used is retention time prediction. In DDA and DIA, peptides are separated on an LC column after sample preparation and digestion. The retention time of a peptide is the time at which it elutes from the LC column material so that the peptides can be recorded by the instrument [16]. The retention time of peptides is determined by their interaction with the stationary and mobile phases of the LC column. Their interaction is determined by their physicochemical properties (e.g., hydrophobicity). A study by Schliekelman and Liu [12] has shown that peptides with very high retention times have a higher probability of being detected than those eluting with several peptides at the same time.

The part of the MS workflow that has the most potential for ML and DL is the prediction of MS spectra from peptide sequence. In multiple experiments, millions of MS/MS spectra can be generated, which is the basis for successful DL approaches [16]. The rapid adoption of DIA as a preferred technique was primarily driven by its ability to provide greater proteomic depth and higher reproducibility across samples compared to DDA. A major catalyst for this shift was the development of advanced software tools like DIA-NN, which utilized DL-based scoring to better navigate the complexity of DIA spectra [39].

While the more recent ability of DL models to predict highly accurate in silico spectral libraries has further enhanced DIA by enabling library-free workflows, these advancements represent a subsequent evolution of the field [31]. The predicted spectra can be used to facilitate protein identification in DDA and DIA analysis, as well as to speed up database searching and in silico spectral library prediction. In silico spectral library prediction has been the cornerstone of DIA analysis since researchers no longer had to rely on spectral libraries from DDA experiments, reducing time and cost [41]. Several applications have been released [27–32]. As shown by Lou et al. [42], the most prominent are DIA-NN [39], Spectronaut [40], and MaxDIA [43].

**Fig. 2.**
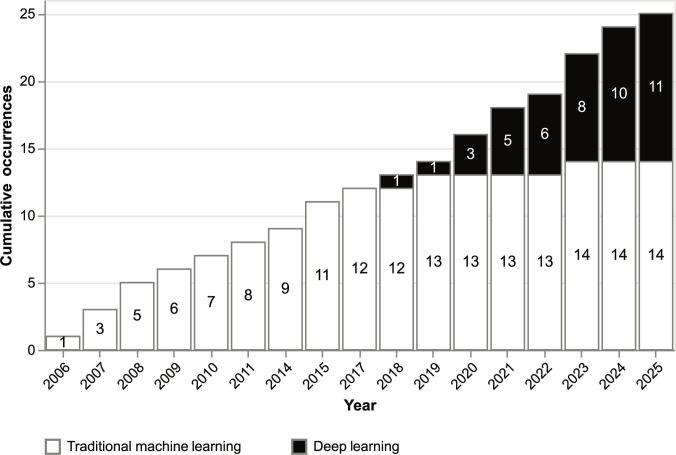
Proportion of traditional machine learning (ML) techniques vs. deep learning (DL) techniques in the field of peptide detectability over time in years starting from 2006 to 2025. From 2006 to 2017, only traditional ML techniques have been published. Since 2018, DL techniques have taken over, and at the time of writing, only a small number of tools released since then apply traditional ML.

Other applications of ML and DL in proteomics include de novo peptide sequencing, prediction of posttranslational modifications (PTMs) of proteins, prediction of protein structure, prediction of major histocompatibility complex-binding peptides, and peptide detectability and more [16]. In this study, we focus on peptide detectability, which will be discussed in more detail below. However, as shown in Fig. 2, it is evident that DL has been used more frequently than ML since 2018. The observed linear trend highlights the steady and continuous growth.

### Peptide detectability in proteomics

Peptide detectability is defined as the probability of detecting a peptide from a solution containing proteins in a mass spectrometry workflow. Most studies investigating peptide detectability approach the problem by modeling it as a binary classification task. The general consensus in the proteomics community is that detectability of a peptide inherently depends on the physicochemical properties of the peptide. However, evidence shows that other factors influence detectability as well [44–46]. Figure 1 presents a standard workflow for tandem MS measurement of a sample in 5 steps, each of which can affect the detectability of peptides. All of these steps are subject to uncertainty and incompleteness. This is illustrated by the fact that, on average, up to 75% of the acquired MS/MS spectra for shotgun proteomics datasets remain unidentified, a phenomenon often referred to as the “dark matter” of proteomics [47]. Below, we highlight some of the most significant limitations of each step.

First, protein extraction efficiency varies across the proteome due to differences in solubility and cellular compartmentalization. Challenges in solubilizing membrane proteins or removing interfering matrix components can lead to incomplete recovery, which subsequently limits the accessibility of the substrate for enzymatic proteolysis [46].

Second, during the cleavage and digestion process, the choice of enzyme is a critical factor. Different proteases exhibit varying rates of missed cleavages, which can influence peptide detectability [44]. Trypsin is the enzyme most commonly used in mass spectrometry due to its high specificity and relatively low rate of missed cleavages, making it the preferred choice for proteomics workflows [45]. The extent of missed cleavages also depends on the digestion conditions. For instance, an extended trypsin digestion typically results in a lower number of missed cleavages, thereby enhancing peptide identification robustness.

Third, LC serves as the primary method for peptide separation prior to mass spectrometric analysis. Coelution of peptides has been observed to negatively impact detectability, likely due to competition during the ionization process. This competition can lead to the undersampling of low-abundance ions, particularly in DDA mode, where precursor selection in MS1 is based on intensity. In DIA, this effect is mitigated to some extent, as all ions are fragmented without precursor selection. However, this effect still contributes to increased spectral complexity, which can affect peptide identification.

Fourth, within the mass spectrometer, both ionization efficiency and the sensitivity of the mass analyzer are key determinants of peptide detectability. In DDA, the selection window for precursor ions inherently biases peptide detection, as only ions within a specific *m*/*z* range are selected, which can systematically exclude certain peptides.

Fifth and last, data analysis begins with raw data processing, where weak or noisy signals may be lost if intensity thresholds are set too high, posing a particular challenge for low-abundance peptides. These thresholds also influence database searching and peptide identification: overly stringent thresholds reduce identification rates, whereas lenient thresholds increase the risk of false identifications. The choice of protein database or reference proteome further impacts identification accuracy, as low-quality references can lead to incorrect or missed identifications.

Considering missed cleavages during data analysis can enhance peptide identification, particularly in relation to sample preprocessing conditions. Since an extended trypsin digestion reduces the number of missed cleavages, this factor should be taken into account to optimize identification rates.

PTMs present an additional challenge in peptide detectability, as they do not appear in the primary AA sequence and must be specifically accounted for during data analysis. Unidentified or unconsidered PTMs can lead to missed identifications or altered fragmentation patterns, further complicating peptide detection.

ML and DL are used to calculate and predict peptide detectability using sequence information, physicochemical properties of the peptides, concentration, digestibility, retention time, and intrinsic ionization properties of the peptide [8,13,14,48–50]. Most of the published techniques that use physicochemical properties of peptides to predict detectability refer to the AA Index database [51]. Researchers agree that the physicochemical properties of each peptide influence its detectability and have shown that ML and DL models can use them to predict peptide detectability [10,11,48,52–61]. Physicochemical properties such as hydrophobicity, charge, and structural state of a peptide can serve as indicators of various properties, including the number of methionine residues, isoelectric point, and prevalence of beta sheets [14,62]. With recent advances in natural language processing, newer approaches have incorporated long short-term memory (LSTM) networks and attentional layers. As shown in Tables 1 and 2, researchers have increasingly relied on peptide sequence information rather than directly using physicochemical properties as features. The rationale behind this shift is that physicochemical properties are inherently encoded in the AA sequence and can be effectively learned by a model given sufficient data, potentially resulting in a better representation of peptide detectability.

**Table 1.** List of traditional machine learning techniques that can predict peptide detectability from 2006 to 2025. The techniques are characterized by the type of technique used, the specific algorithm used in the paper, and the properties used for prediction. They are listed from the oldest publication date to the newest.

Year	Technique/Paper	Technique detailed	Properties used for prediction
2006	A computational approach toward label-free protein quantification using predicted peptide detectability [9]	MLP (multilayer perceptron)	Sequence, physicochemical properties
2007	Prediction of peptides observable by mass spectrometry applied at the experimental set level [55]	MLP	Physicochemical properties
2007	PeptideSieve [58]	Gaussian mixture likelihood function	Physicochemical properties
2008	APEX [11]	RF (random forest) classifier	Physicochemical properties
2009	ESPPredictor [59]	RF classifier	Physicochemical properties
2010	The importance of peptide detectability for protein identification, quantification, and experiment design in MS/MS (tandem mass spectrometry) proteomics [52]	MLP	Physicochemical properties
2010	A support vector machine model for the prediction of proteotypic peptides for accurate mass and time proteomics [57]	Support vector machines	Physicochemical properties
2011	CONSeQuence [10]	Ensemble of different models (RF classifier, support vector machine, MLP, genetic programming)	Physicochemical properties
2014	PeptideRank [53]	Ensemble of different models (regression trees, MLP)	Physicochemical properties
2015	PPA [94]	MLP	Physicochemical properties
2015	PREGO [33]	MLP	Physicochemical properties
2017	Enhanced missing proteins detection in NCI60 cell lines using an integrative search engine approach [49]	RF classifier	Physicochemical properties
2019	AP3 [60]	RF classifier	Missed cleavage, sequence, physicochemical properties
2023	PeptideRanger [56]	RF classifier	Physicochemical properties, missed cleavage

**Table 2.** List of deep learning techniques that can predict peptide detectability from 2006 to 2025. The techniques are characterized by the type of artificial intelligence used, the specific algorithm used in the paper, and the properties used for prediction. They are listed from the oldest publication date to the newest.

Year	Technique/Paper	Technique detailed	Properties used for prediction
2018	d::pPop [48]	FNN (feed forward neural network)	Physicochemical properties
2020	DeepMSPeptide [50]	CNN (convolutional neural network)	Sequence
2020	DeepDIA [63]	Hybrid neural network (CNN, bidirectional long short-term memory network [bi-LSTM])	Sequence
2021	PepFormer [8]	Transformer and GRUs (gated recurrent units)	Sequence
2021	CapsNet [54]	Capsule neural network	Physicochemical properties, sequence
2022	PD-BertEDL [61]	Bi-LSTM	Physicochemical Properties, sequence
2023	DeepDetect [13]	Bi-LSTM	Missed cleavage, sequence
2023	DbyDeep [64]	LSTM	Missed cleavage, sequence
2024	KDEAN [65]	Bi-LSTM	Sequence
2024	DeepPD [66]	Transformer	Sequence
2025	Pfly [67]	Bi-recurrent neural networks (BRNN) and GRU	Sequence

#### The conceptual framework of peptide detectability

Peptide detectability is frequently treated as a binary classification task in ML, the physical reality on the other hand is more complex than that. We distinguish between 3 different concepts which are “Flyability”, “Observability”, and “Detectability”. These terms describe distinct stages of the analytical chain, and conflating them can lead to a misunderstanding of what a model is truly learning. Flyability refers to the ionization efficiency and gas-phase transportation within the MS. It is an intrinsic property of the peptide and ionization source. Observability is a broader, context-dependent term that encompasses the entire experimental window, including the peptide’s presence in the sample after digestion, its recovery during chromatography, and its concentration relative to the instrument’s dynamic range. Detectability is often used in the literature as a synonym for the above terms, though it specifically describes if the detector can register a specific peptide or not. A core issue that joints the above-described conceptual issue is the definition of the nondetectable class. A peptide labeled as nondetectable can represent 3 phenomena. The first is the biological absence of the protein and therefore the peptide within the sample. Second to that is the technical absence, which means the peptide is present but failed to ionize, was lost during sample preparation, or fell below the detection limit. The third is the sampling absence. In DDA experiments, it can happen that the peptide was available and detectable but was not selected for fragmentation due to stochastic undersampling. The definition of “detected” is further filtered through bioinformatic choices. False discovery rate (FDR) thresholds used during search engine processing directly dictate which peptides are assigned positive labels. Furthermore, while DIA workflows reduce sampling absence, they introduce new challenges in signal deconvolution that can lead to false negatives. Finally, it is important to note that the target variable varies across existing tools. While some utilize a binary classification (observed vs. unobserved), others treat detectability as a continuous probability or use MS1 signal intensity (abundance) as a proxy. This lack of a standardized “ground truth” remains a core challenge for cross-tool performance comparison and reproducibility.

By acknowledging these nuances, it becomes clear that “detectability” models are not predicting a physical constant, but rather the probability of a peptide surviving the entire analytical pipeline under specific instrumental conditions and selection of parameters like FDR filtering in postprocessing steps.

#### Overview of the analyzed tools

**Fig. 3.**
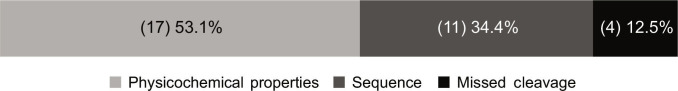
Proportion of input features for different machine learning techniques from 2006 to 2025. About half of the tools use physicochemical properties, a third use amino acid sequence information, and ~13% also use missed cleavage as a feature. Techniques that use multiple input features contribute to each relevant category in this graph, so a single technique may be represented in more than one feature proportion.

**Fig. 4.**
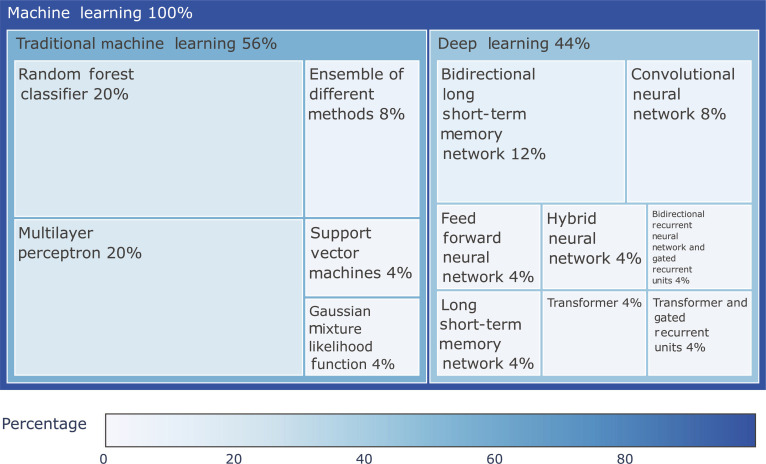
This treemap shows the distribution of machine learning techniques in proteomics, divided into traditional (left) and deep learning (right) techniques. The area of each rectangle reflects the occurrence of the technique in publications from 2006 to 2025. Among traditional techniques, random forest and multilayer perceptron show significant representation, while in deep learning, bidirectional long short-term memory networks dominate, highlighting a shift toward deep learning in recent years.

Since the first publication in 2006 [9] of an ML approach using a multilayer perceptron (MLP), over 25 technique papers have been published on peptide detectability. Figure 3 shows that more than half of the techniques used physicochemical properties. Almost all traditional ML techniques used physicochemical properties as input features to predict peptide detectability. Representing peptides by their physicochemical properties allows the classification of detectable and nondetectable peptides. However, this approach has shortcomings. For example, 2 peptides with the same AA composition but different sequences may have similar calculated physicochemical properties despite different structures and detectability [8]. Sequence-based techniques, used by a third of the techniques analyzed (Fig. 3), address this by encoding the entire sequence, taking into account the AA order. Four techniques included missed cleavage prediction (Fig. 3) to account for imprecise enzyme reaction, with ML predicting missed cleavage for more accurate experimental conditions. The 2 most common techniques for peptide detectability have been the random forest classifier and MLP, shown in Fig. 4, which uses numerical features representing physicochemical properties and offers satisfactory performance and interpretability. In addition, MLP and support vector machines have been used. Since 2018, DL techniques have been used more extensively than traditional ML techniques. The growing prevalence of DL techniques since 2018 is likely the result of multiple factors. Advances in hardware, particularly in graphical processing units, have enabled the training of larger and more computationally intensive models. In parallel, developments in natural language processing have led to new architectures and embedding strategies, encouraging researchers to represent AA sequences as input directly rather than relying solely on predefined physicochemical descriptors. This shift is based on the assumption that sequence-based representations may better capture structural or contextual features relevant to peptide detectability. However, a direct connection between DL approaches and improved predictive performance cannot currently be established, due to the lack of standardized benchmarks and differences in datasets, model architectures, and evaluation protocols across studies. These limitations are discussed in more detail in a later section. A controlled comparison across ML and DL models would be needed to draw reliable conclusions about relative advantages. Protein abundance and its influence on peptide detectability are discussed in depth in 9 of the 25 analyzed papers, yet it is only incorporated as an input feature in one method: the PPA classifier. DDA-based datasets are based on the selection of the most abundant precursors, which introduces a bias when it comes to the topic of detectability. Using DIA-based datasets, which capture a broader dynamic range, may mitigate this limitation by reducing dependency on abundance alone for detectability. PTMs and their impact on detectability are addressed in detail in 3 of the 25 papers. However, none of the classifiers incorporate PTM information as an input. Additionally, none of the datasets used include MS data in which PTMs were systematically identified. Since PTMs can impact peptide ionization and fragmentation—and thus, detectability—future work could benefit from predicting potential PTMs from sequence data. Incorporating these predictions into model inputs could improve accuracy and biological relevance. However, generating training data for PTMs remains challenging, as nondetectable PTMs are inherently difficult to identify. Unlike nondetectable peptides, their absence cannot be reliably inferred from the data. In addition to differences in methodology, the extent to which tools are actively maintained also varies considerably. To provide insight into the current relevance and sustainability of each tool, we include an overview of their most recent updates in Table S1. The term “Unknown” has been put if the tools were not available or no update notes could be found. Recently, natural language processing techniques such as bidirectional long short-term memory (bi-LSTM) networks and Transformer models using sequential peptide inputs have become prominent.

The overall distribution of most techniques up to 2018 also suffered from poor reproducibility. Tables 1 and 2 show that except for the APEX [11] tool, the source code of the other tools or the data they used in the paper are not available. In addition to the hand-picked datasets, this makes it difficult to compare the results of these papers. For the following comparison of detectability tools using ML, we used only some of the tools with greater reproducibility and usability.

#### Comparison of the tools

From the 25 tools represented in Tables 1 and 2, we selected 10 that can be viewed in Table 3 based on the availability of source code, data, and reports of ML performance metrics in peer-reviewed publications. The papers by Braisted et al. and Yang et al. [11,63] and by Searle et al. [33] were excluded from further consideration because they did not report performance scores for peptide detectability. We briefly describe the selected techniques, focusing on their training data, feature representations, reported performance, and evaluation strategies. These comparisons are summarized in Table 3.

**Table 3.** List of selected techniques describing the type of artificial intelligence used by the authors, whether they reported a training/test/validation split of their data, which organisms they used, the size of the training set used to train their model, and the performance metrics they reported.

Year/Technique	Train, test, validation split	Organisms used for training	Training set size (number of peptides	Class imbalance detectable/nondetectable in %	Reported performance metrics
2019/AP3 [60]	Yes	Mouse, bacteria, human, yeast	~43,000	50/50	AUC (area under the curve)
2020/DeepMSPeptide [50]	No	Human	Not defined	Unknown	AUC, ACC (accuracy), SP (specificity), SN (sensitivity), F1 score
2021/PepFormer [8]	Yes	Human, mouse	~70,000	50/50	ACC, SP, SN, MCC (Matthews correlation coefficient)
2021/CapsNet [54]	Yes	Various organisms (not specified)	~90,000	Unknown	AUC, ACC, SP, SN, F1 score
2023/DeepDetect [13]	Yes	Human, yeast, bacteria	~400,000	50/50	AUC
2023/DbyDeep [64]	Yes	Human, mouse, yeast	~500,000	50/50	AUC, ACC, SP, SN, precision
2023/PeptideRanger [56]	Yes	Human	10,000	50/50	AUC, ACC, SP, SN, precision, F1 score
2024/KDEAN [65]	Yes	Human and mouse	~90,000	50/50	AUC, ACC, F1 score, MCC
2024/DeepPD [66]	Yes	Human and mouse	~90,000	49.41/50.59	AUC, ACC, F1 score, MCC
2025/Pfly [67]	Yes	Human	~187,000	50/50	AUC, ACC, recall, precision, F1 score, MCC

The first tool is AP3 [60], which employs a random forest classifier using physicochemical properties and predicted peptide digestibility as input features. A total of 588 features were used to characterize each peptide. The model was trained on 3,959 yeast proteins and validated on *Escherichia coli*, mouse, and human datasets. The authors demonstrated that digestibility was the most influential feature in the model, highlighting the strong mechanistic dependence of peptide detectability on enzymatic cleavage. The peptide detectability model with 29 selected features achieved a 10-fold cross-validation area under the curve (AUC) of 0.9269 on yeast, compared to 0.8891 without peptide digestibility. AP3 reportedly outperformed PeptideSieve [58], CONSeQuence [10], and ESPPredictor. However, all benchmarks were conducted using an in-house dataset, limiting external validity and introducing potential evaluation bias.

DeepMSPeptide [50] uses a convolutional neural network (CNN) to predict peptide detectability from AA sequences alone. Training and evaluation data were derived from the Global Proteome Machine Database (GPMDB, https://thegpm.org/), though the authors did not clearly delineate training, validation, and testing splits. A total of 24,000 peptides were used for benchmarking: 8,000 peptides from detected proteins, 8,000 from proteins missing in 2011, and 8,000 from proteins still missing. The CNN achieved an AUC of 0.86, outperforming prior feature-based models [48,49,58]. However, it was not benchmarked directly against AP3, which integrates digestibility and showed strong performance in targeted proteomics settings. Furthermore, class balance and organism-specific generalizability were not explicitly discussed.

PepFormer, developed by Cheng et al. [8], utilizes a modular architecture including a sequence embedding model, a context-sensitive encoder, and a bidirectional gated recurrent unit (GRU) to capture positional dependencies. A Siamese network with contrastive loss is used for binary classification. The model was trained on *Homo sapiens* and *Mus musculus* peptides from GPMDB using an 80%/20% training/testing split and benchmarked against AP3 [60] and DeepMSPeptide [48,50]. Standard metrics including accuracy (ACC), specificity (SP), sensitivity (SN), and Matthews correlation coefficient (MCC) were reported. Although PepFormer demonstrated improved cross-species generalization, its comparisons relied on reimplemented baselines rather than original source code, limiting strict reproducibility. The authors also stated that source code for prior tools was unavailable, which could not be independently confirmed.

CapsNet, proposed by Yu et al. [54], introduces a capsule network with a convolutional block attention module (CBAM) for peptide detectability prediction. The model uses 4 feature types derived from the peptide sequence: residue conical coordinates, AA composition, dipeptide composition, and a sequence embedding code. These are grouped into biological and sequence features and processed through parallel CapsNet branches with CBAM assigning channel- and spatial-wise attention before the capsule layers. Training was performed on approximately 90,000 tryptic peptides from GPMDB originating from multiple organisms, using 10-fold cross-validation as well as an additional 10,000-peptide subset split 75%/25% into training and test sets. On the GPMDB test set, CapsNet achieved an AUC of 0.8692 and an accuracy of 0.8063, outperforming the 1D-2C-CNN architecture of DeepMSPeptide and several classical ML baselines across AUC, ACC, SN, and F1 score. The authors also evaluated the model on Human Proteome Project benchmark sets and on 2 therapeutic peptide datasets (antiangiogenic and antibacterial peptides), demonstrating that the architecture can generalize to related peptide classification tasks, although all evaluations relied on hand-crafted feature encodings rather than purely sequence-based representations.

DeepDetect [13] combines a bi-LSTM network for peptide sequence modeling with predicted digestibility from DeepDigest [18]. The square root of the product of both outputs is used as the final detectability score. Seven human and one yeast dataset were used, comprising 55,261 proteins digested using multiple enzymes. By integrating digestibility directly into a DL framework, DeepDetect reflects the experimentally causal relationship between cleavage and detection. However, the authors retrained PepFormer for comparison because the original model was limited to tryptic peptides, which reduces the fairness of the comparison. In addition, the comparative evaluation focuses almost exclusively on receiver operating characteristic (ROC) AUC, and other metrics such as accuracy or MCC are not systematically reported.

DbyDeep [64] builds on a similar framework by integrating peptide sequence information with cleavage site context through 3 modules: an LSTM-based sequence encoder, a cleavage context module, and a fully connected classifier. The dataset consists of human peptides, divided into training (536,492 peptides), validation (134,124 peptides), and test (170,367 peptides) sets. Evaluation relied primarily on AUC across human, yeast, and mouse datasets. While DbyDeep consistently outperformed competing tools in the reported benchmarks, all baseline models were retrained on the same dataset without detailed hyperparameter optimization. Consequently, the improvements—often on the order of ∆AUC ≈ 0.01—may not be statistically meaningful. No formal significance testing was reported, limiting confidence in claims of superiority.

PeptideRanger [56] is an R package based on a random forest classifier trained on 75 physicochemical features. The primary dataset consisted of 20,373 human proteins from ProteomicsDB (https://www.proteomicsdb.org/), with 10,000 peptides used for training and 50,000 used for testing. A second reduced-feature model was generated using the top 15 ranked features. Two benchmarking strategies were employed: one using ProteomicsDB data only, and a second using independent data from CPTAC and PeptideAtlas (https://pdc.cancer.gov/ and https://peptideatlas.org/). Potential dataset bias exists in the first evaluation, as PeptideRanger was trained exclusively on ProteomicsDB data, unlike the baseline models.

KDEAN [65] applies a bi-LSTM with an external attention mechanism to learn bidirectional peptide embeddings. Three networks encode AA sequence, AA composition, and structural context, with outputs combined via element-wise summation and a sigmoid-activated classifier. The model was trained on 4 datasets (3 human and 1 mouse). Each dataset was randomly split 8:2 into training and test sets, and 10% of the training set was held out as a validation set. Benchmarking against AP3, DeepMSPeptide, PepFormer, and DeepDetect showed only marginal improvements over DeepDetect across accuracy, F1 score, MCC, and ROC AUC.

DeepPD [66] integrates multiple sequence representations for peptide detectability, including BLOSUM62 encoding, word-embedding-based 2D feature maps, and contextual embeddings from the protein language model ESM-2. BLOSUM62 and the 2D feature map are processed through 1D CNNs and Bi-GRU layers, while the ESM-2 embedding is reduced in dimensionality. The combined representation is passed to a fully connected layer for binary classification. Training was conducted on the reorganized DDA dataset from Ref. [8] comprising approximately 70,000 peptides. While the use of protein language models represents a methodological advancement, external validation and multiorganism generalization were not extensively explored.

Pfly [67] is the most recent tool, utilizing an encoder–decoder architecture with an attention mechanism and a bidirectional GRU to classify peptides into nonflyers, weak, intermediate, and strong flyers; the 3 flyer classes are aggregated to obtain a binary detectability score. The base model was trained on 328,832 unique tryptic peptides from the ProteomeTools synthetic peptide library, split 90:10 into training and test sets, with 20% of the training data reserved for validation. To mitigate biases toward synthesizability, the authors then fine-tuned Pfly on a large in vivo DDA dataset from 6 human cell lines digested with 6 proteases [67]. For external evaluation, Pfly and several baselines (PepFormer, DeepMSPeptide, DeepDetect, and PeptideRanger) were benchmarked on an independent human tissue dataset by Wang et al. [68] and on a cross-species dataset, reporting ACC, F1 score, MCC, and AUC. While Pfly consistently outperformed the reimplemented baselines on these benchmarks, it is important to note that the competing models were (re)trained or executed under the authors’ own settings, and their performance metrics do not necessarily match those reported in the original publications. Moreover, relying on synthetic peptides for initial training introduces the potential for bias toward synthesis. Although the authors discuss and partially address this issue by fine-tuning empirical DDA data, the extent to which synthetic bias persists in highly complex, endogenous samples remains a limitation that requires independent benchmarking.

Overall, a methodological trend emerges across these tools: models that explicitly incorporate peptide digestibility (AP3, DeepDetect, and DbyDeep) improve their predictive capabilities, underscoring enzymatic cleavage as a supporting determinant of detectability. Similarly, recent tools increasingly leverage multimodal representations and large-scale pretraining (DeepPD and Pfly), improving generalization at the cost of reduced interpretability and increased computational complexity. Despite steadily improving AUC and MCC values across studies, direct performance comparisons remain difficult due to heterogeneous datasets, reimplementation of baseline models, inconsistent reporting of class balance, and the absence of statistical significance testing. These factors highlight the continued need for standardized benchmarking frameworks and shared reference datasets for peptide detectability prediction.

The selected techniques have publicly available source code and data. The publication dates of the techniques range from 2019 to 2025. Based on the publication dates, it can be observed that the reproducibility and applicability of the papers in the field of peptide detectability have increased over time.

Figure 2 and Tables 1, 2, and 3 show that from 2018 onward, only 2 instances of traditional ML techniques were reported, while the rest utilized DL. This illustrates the rise of deep neural networks and correlates with the increasing amount of available mass spectrometry data. Although DL architectures possess immense representational capacity, there is currently no definitive evidence establishing that they provide consistent performance gains over traditional algorithms for peptide detectability prediction. The choice between traditional models and DL must be strictly guided by the relationship between model complexity and available dataset size. DL requires large training datasets with high sample complexity to generalize effectively and avoid overfitting. In scenarios characterized by limited data availability, traditional tree-based algorithms, such as random forests or gradient boosting machines, can outperform DL approaches.

Furthermore, the transition to DL introduces significant trade-offs regarding computational cost, energy footprint, and model interpretability. DL models demand substantial computational resources, frequently requiring specialized graphics processing units for both extensive training cycles and routine inference. This high energy footprint can severely limit the practical deployment of these tools in resource-constrained laboratory environments. Conversely, traditional ML models are computationally lightweight and can be executed efficiently on standard hardware. Finally, the inherent opacity of deep neural networks presents a significant barrier to biological understanding. While deep neural networks often operate as black boxes, tree based models offer intrinsic interpretability through accessible feature importance metrics. This transparency allows researchers to more easily identify the specific physicochemical properties that influence detectability. Therefore, while DL pushes the boundaries of predictive scale on massive datasets, traditional ML remains an indispensable framework for tasks that prioritize interpretability, computational sustainability, and robust performance on smaller datasets.

This divergence in algorithmic complexity is closely mirrored by a fundamental shift in how peptide data are represented as input. Researchers have shifted from primarily using physicochemical properties to peptide sequences. This shift in data representation can be attributed to the demonstration in several techniques outlined in Table 3 that physicochemical properties are encoded in the peptide sequence. The advent of LSTM and bi-LSTM networks has enabled the improvement of peptide sequence feature extraction, establishing a reliable approach that does not rely on hand-picked physicochemical properties as input. Apart from AP3 and DeepDetect, researchers have reported multiple performance metrics, increasing the transparency of the evaluation process. Selecting a specific metric, such as AUC, may bias the reader and not fully represent the performance of the classifier. While none of the current tools can be universally recommended as optimal, some may be better suited depending on the user’s priorities and the characteristics of their dataset. Tools that prioritize ease of deployment, open-source availability, and user-driven fine-tuning options (e.g., Pfly) offer distinct practical advantages. However, independent comparative benchmarking is still required to definitively determine the superior tool for specific experimental workflows.

### Guidelines for the application of AI standards in the field of peptide detectability

After exploring the potential of peptide detectability, we compared the most usable and reproducible techniques and found that while reproducibility and ML standards have improved over time, many studies still fall short of established practices in the ML community. However, some recent methodologies demonstrate improved alignment with these standards; for instance, models like Pfly have begun to explicitly address data access, cross-dataset validation, class imbalance, and model generalization through synthetic pretraining and fine-tuning strategies [67]. The number of different datasets used for training, testing, and validation makes it difficult to directly compare the performance of tools. To provide guidelines, we have used the paper by Heil et al. [69], Walsh et al. [70], and Palmblad et al. [71] as reference.

**Recommendation 1.** Ensure the availability of the source code, trained ML models, and raw data used in the study by including a dedicated section in the paper. This practice is particularly important in the field of proteomics, where the selection of datasets strongly influences the generalizability and performance of ML models. We recommend using a cloud-based repository manager for easy access to the source code. The user can provide detailed documentation of the tool there to increase usability and reproducibility.

**Recommendation 2.** Add a section describing the data in detail. This includes a description of the data acquisition, sample preparation, preprocessing, data type, and format of the data. Further details on the potential data imbalance between detectable and undetectable peptides are missing. In addition, it should be clear whether the authors classify peptide sequences as undetectable when they correspond to proteins that are expected to be present in the sample but for which no peptides were detected. Further, as pointed out by Palmblad et al. [71], it should also be stated whether the classifier is applicable to all peptides or is limited for example to tryptic peptides.

**Recommendation 3.** The data should be split into training, test, and validation sets, well described, and cross-validated. For example, common split ratios are 60%–20%–20% or 70%–15%–15%. In particular, the direct input into the ML models is important to emphasize for traceability.

**Recommendation 4.** Use multiple performance metrics to transparently show how the tool performs and where it might be better or worse than other tools. This is essential for transparency. Since peptide detectability is usually a binary classification task, we recommend using sensitivity and specificity to show the true positive and true negative rates. The F1 score shows how well the classifier can predict both classes, detectable and nondetectable. This is important because it takes into account class imbalance and informs the user whether the classifier can accurately predict both classes. To ensure clarity, it is critical to explicitly state which class is treated as the positive class. In most peptide detectability studies, this refers to the detectable class. However, depending on how the dataset is constructed, the positive class may not always be the minority class. This can distort metrics such as precision, recall, and F1 score if not clearly defined. Therefore, we recommend reporting the class distribution and clearly denoting which class was considered positive in all evaluations. The ROC curve can be used as a visual identification of classifier performance. It is generated by plotting the sensitivity against the false positive rate. The closer the plotted curve is to 0 and 1, the better the classifier is [72]. The MCC should also be reported as it will only produce a high score if the classifier performs well for true positives, false negatives, true negatives, and false positives [73]. This is also recommended in DOME (data, optimization, model, evaluation) guidelines [70].

**Recommendation 5.** While recent tools often report marginal performance improvements over baseline models, these claims are frequently made without assessing statistical significance. To ensure methodological rigor, we recommend that future studies adopt context appropriate statistical testing to determine whether minor performance gains are genuine rather than artifacts of data splitting. When comparing predictions from a single independent test set, the DeLong [74] test is explicitly recommended for comparing ROC curves, while McNemar’s test [75] should be utilized to compare the sensitivity or specificity of binary predictions. Conversely, when comparing models across multiple folds or resampled test sets, standard paired *t* tests should be avoided, as they are sensitive to outliers and assume data independence that resampling methods inherently violate. Instead, the nonparametric Wilcoxon signed-rank test [76] should be used to compare 2 models, whereas Friedman’s test [77] is required when comparing 3 or more models simultaneously to prevent the inflation of Type I errors caused by multiple pairwise comparisons. Furthermore, evaluating the variance and stability of a model across different datasets is just as critical as its median performance. Because performance metrics such as accuracy or AUC are bounded between 0 and 1, their distributions are often skewed and nonnormal. Consequently, instead of standard *F* tests, researchers should apply the Shapiro–Wilk [76] test to verify normality, followed by Levene’s test [78] or Bartlett’s test [79] to rigorously compare model variance. The article on evaluation metrics and statistical tests for ML by Rainio et al. [72] has been a great resource for our guideline.

Beyond statistical significance, evaluations must clearly distinguish between threshold-independent metrics (e.g., AUC ROC and area under the precision recall curve [AUC PR]) and threshold-dependent metrics (e.g., Accuracy and F1 score). Because peptide detectability datasets can be imbalanced, with nonflyers outnumbering flyers, AUC ROC can present an overly optimistic view of performance. The AUC PR should therefore be reported as a more informative, threshold-independent alternative. Furthermore, when reporting threshold-dependent metrics, authors must transparently justify their chosen decision boundary rather than relying solely on default values. Additionally, models should be evaluated on their calibration, which measures how well the predicted probabilities reflect the true frequency of observation. A model may achieve a high AUC while being poorly calibrated, rendering its probabilistic outputs unreliable for downstream experimental planning. We recommend reporting calibration metrics such as the Brier score [80] or expected calibration error (ECE). Finally, because models trained predominantly on synthetic peptide libraries frequently suffer performance degradation when applied to different contexts, robust evaluation must explicitly assess out-of-distribution generalization. This should be achieved through zero shot testing on independent datasets to evaluate domain shift, utilizing cross-instrument, cross-protocol, or cross-species benchmarks.

**Recommendation 6.** Show in detail the hyperparameters used in model selection and how they were determined. This includes what type of encoding or embedding has been used for the input data. For the classifier that utilizes physicochemical properties, it is important to show which have been used as input data. This is especially important if not all calculated physicochemical properties were used and explains why they were selected. Additionally, the authors should provide training and inference time and discuss the interpretability of their model and if explainability of predictions is provided [70,81,82].

**Recommendation 7.** Provide the metadata for the data utilized in training, validating, and testing the classifier. Especially for the task of peptide detectability, it is important to document the reference database and search engines, as they have a big influence on the final detectability. It is also important to mention the details about sample preparation, microbial cultivation, and mass spectrometer parameters.

**Recommendation 8.** Sequence similarities between species used for training, testing, and validation as well as technical confounding factors should be carefully assessed to prevent data leakage. To effectively mitigate biological leakage, dataset partitioning should strictly employ protein level splitting rather than peptide level splitting, and homology clustering must be performed prior to division. The software suite *MMseqs2* is well-suited for large-scale datasets, such as those frequently used in ML studies on peptide detectability, and can efficiently identify highly similar AA sequences across species and datasets [83]. For example, the human and mouse genomes are highly similar, each containing approximately 30,000 protein-coding genes, with around 80% having identifiable orthologs in the other species, which makes data leakage between these datasets a considerable risk [84]. Furthermore, evaluations must account for shared proteotypic peptides across orthologs in multiorganism datasets. Finally, to ensure real-world generalizability, cross-validation strategies should incorporate independent instrument batches and temporal holdouts. Addressing temporal leakage is particularly critical because hardware advancements shift the data quality. Modern mass spectrometers capture greater proteomic depth, meaning peptides considered nondetectable in older datasets may be detectable today.

Taken together, these recommendations define a set of good practices for the development and reporting of AI-based peptide detectability models. While many of the reviewed studies only partially adhere to these standards, recent literature demonstrates that comprehensive compliance is both feasible and beneficial. Implementing public code availability, large-scale pretraining, systematic fine-tuning on diverse *in vivo* datasets, and explicit treatment of class imbalance illustrates that rigorous AI standards do not constrain methodological innovation. Rather, they enable more robust, transparent, and transferable peptide detectability prediction. We therefore encourage future studies to adopt these guidelines not only to improve reproducibility and comparability, but also to accelerate the practical integration of peptide detectability models into experimental proteomics workflows.

## Materials and Methods

### Search strategy

**Fig. 5.**
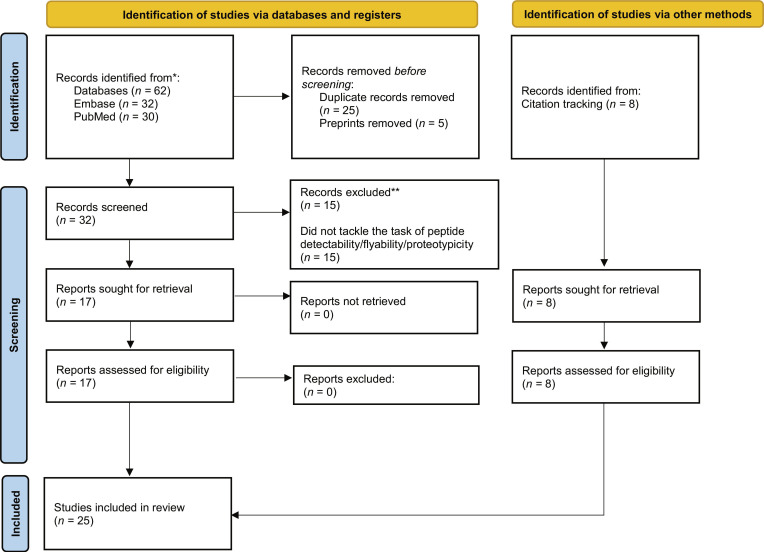
Flow diagram of the scoping review, adapted from the Preferred Reporting Items for Systematic Reviews and Meta-Analyses (PRISMA), showcasing the literature search and the identification of articles. Articles were identified (*n* = 62), after deduplication (*n* = 37), after removal of preprints (*n* = 32), after title/abstract screening (*n* = 17), and after citation tracking (*n* = 25).

To ensure methodological transparency, the literature identification and selection process was documented using a flow diagram in Fig. 5.

The literature search was performed using the Ovid research platform, with PubMed and Embase selected as the primary databases. The search targeted the titles and abstracts of research papers. The core search string, which identified 61 initial studies, was structured as follows:


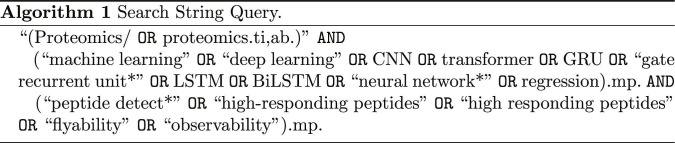


### Inclusion and exclusion criteria

Articles were evaluated against predefined inclusion and exclusion criteria. Studies were included if they met all of the following requirements:

1. Original research articles published between 2000 January 1 and 2025 November 13.
2. Studies that explicitly addressed the topic of peptide detectability using techniques from the field of ML and DL.
3. Studies that specifically created their own stand-alone predictor or implemented one within a larger framework.
4. Studies written in English.

We followed the following criteria for the exclusion of articles.

1. Studies lacking an ML or DL component.
2. Studies that are not peer-reviewed and are only available as preprints.

### Study selection

Following the initial database search, deduplication yielded 37 unique records. To ensure that all evaluated algorithmic methodologies and performance metrics had undergone independent scientific validation, preprints were explicitly excluded, leaving 32 candidate articles. Subsequent title and abstract screening led to the exclusion of 15 studies that did not meet the predefined criteria, resulting in 17 articles selected for full-text review. An additional 8 highly relevant studies were identified through backward and forward citation tracking, particularly those utilized as benchmark comparisons within the field. During the full-text evaluation, we did not encounter any borderline cases.

### Quality assessment and guideline synthesis

To assess the methodological quality of the included studies and address observed reporting gaps, papers were evaluated utilizing established ML frameworks mapped to the specific requirements of computational proteomics. The foundational structure of our assessment and subsequent recommendations aligns with the community-wide DOME guidelines for supervised ML in biology, as well as their domain-specific interpretation for mass spectrometry-based proteomics [70,71]. Specifically, recommendations 1, 6, and 7 were adapted from the reporting principles outlined by Heil et al. [69], while recommendations 2 and 4 were informed by the standards proposed by Bender et al. In contrast, recommendations 3, 5, and 8 were developed independently as original contributions of this study. These were formulated based on our systematic quality analysis of the 25 included peptide detectability tools, specifically addressing critical observed gaps in the reporting of dataset splits and the mitigation of sequence-based data leakage.

## Discussion

Peptide detectability has the potential to significantly advance computational proteomics by improving protein quantification, enabling more effective biomarker discovery, and optimizing targeted workflows. By focusing on consistently detectable peptides, researchers can increase experimental design efficiency, reduce complexity, and improve data quality, especially in challenging biological samples. In particular, for species identification in biological samples, accurate prediction of detectable peptides can reduce the search space in large databases such as UniProt, which is critical for DIA data analysis and can reduce processing time. In SRM, MRM, or PRM workflows, selecting the right peptides is often a process of trial and error. Detectability predictors allow researchers to prioritize proteotypic peptides with high ionization efficiency and favorable fragmentation. In label-free quantification methods using either DDA or DIA, a major challenge is distinguishing between a peptide that is biologically absent and one that is simply below the limit of detection or obscured by noise. Incorporating detectability scores as statistical priors allows for the correction of sampling bias and interference. This enables more accurate estimation of protein abundance from sparse peptide observations and helps to intelligently impute missing values based on the likelihood of a peptide being observable. By focusing on peptides with high detectable consistency, researchers can design studies that are less prone to missing values across large patient cohorts. This leads to more robust statistical power in biomarker discovery because the resulting data matrices are inherently more complete.

ML and DL techniques are particularly suited for predicting peptide detectability due to their ability to recognize complex patterns and identify nonlinear relationships within diverse datasets. Nonetheless, these approaches carry inherent limitations, most notably the “black-box” problem. While some traditional ML models offer a degree of interpretability, complex DL architectures often make it exceedingly difficult to understand the exact rationale behind classifying a specific peptide as detectable or nondetectable. Despite this lack of transparency, there are currently no competitive analytical alternatives that can match the predictive scale and performance of AI for this specific task.

However, current peptide detectability models still face barriers to broader adoption, including challenges related to reproducibility, transparency, and data selection. Notably, most existing classifiers are trained and evaluated on DDA data, which are more widely available but introduce potential biases due to their precursor selection strategy that favors high-abundance peptides. As a result, peptides that are consistently detectable in DIA workflows may be underrepresented or indirectly treated as nondetectable due to missing sampling in DDA data. Increasing the use of DIA data for model training and evaluation could help reduce such biases and better reflect the diversity of peptides observed in contemporary proteomics experiments. Facilitating access to high-quality, labeled DIA datasets will therefore be essential to improving the robustness and applicability of peptide detectability models in practice.

After exploring the potential of peptide detectability, we compared the most usable and reproducible techniques and found that while reproducibility and ML standards have improved over time, they still fall short of established practices in the ML community. The variety of datasets used for training, testing, and validation complicates direct performance comparisons between tools. In Results, we identified several key guidelines to align with AI standards in the field of peptide detectability, which also inform and strengthen future research in other areas of computational proteomics.

The first guideline is to include a separate section for tool and data access. For example, Gao et al. [60] provided a brief section describing access to their tool along with a complete user guide. This approach ensures that users can easily find, set up, and use the tool without overwhelming the paper with technical details, thereby improving usability and reproducibility. We recommend providing access to data via publicly available repositories such as Peptide Atlas, MassIVE, PRIDE, iProx, JPOST, or Panorama, and linking via a ProteomeXchange identifier. The ProteomeXchange Consortium [85] consolidates these datasets, ensuring broad accessibility and facilitating proper citation. Inclusion of data used as input to AI models would further enhance reproducibility, allowing researchers to validate the techniques and their performance.

Regarding the second guideline, we recommend including a dedicated section that thoroughly describes the data used. This should encompass a detailed account of data acquisition procedures. For mass spectrometry-based proteomics, this includes information on sample preparation (such as chemicals used, enzymes applied, and incubation times), the type of mass spectrometer, and the *m*/*z* ratio window. These parameters are critical, as they have a significant impact on the characteristics of the resulting data. In addition, the potential imbalance in the raw data between detectable and undetectable peptides should be explicitly acknowledged, given that undetectable peptides typically comprise a much larger portion of the dataset. It should also be specified whether undetectable peptides are defined only for proteins from which at least one peptide was detected, or if they also include proteins for which peptides were expected but not observed. For full traceability and reproducibility, the exact input data fed into the ML model must be clearly described. Furthermore, the type of data acquisition method—whether DDA, SRM, or DIA—should be stated explicitly. While this information is often inferable from the experimental description, providing it directly enhances clarity and transparency. This level of detail is particularly important in cross-disciplinary fields such as mass spectrometry-based proteomics combined with AI, where researchers must assume their work will be read by experts from both domains.

Another section of the paper should directly describe the dataset splits and inputs used for each AI technique. It must be clear what part of the dataset was used as training data, test data, and validation data. In most of the technique papers we covered here, the data were split and cross-validated, but it was often difficult to identify this. In addition, the direct input and output format of the AI model should be reported, for example, in the paper by Gao et al. [60], where they directly stated what physicochemical properties went in as input, or in the paper by Yu et al. [54], who showed that their input is a vector representation of the AA sequence of the peptides. Most of the techniques briefly described what encoding or embedding they used, which is an important factor in the performance of ML models as mentioned by Spänig et al. [86]. It would be interesting to compare different encodings and embeddings for AA on these techniques to see if performance can be improved.

One of the most important things in the development of a new AI technique in proteomics, as in other fields, is its performance. This refers to the guideline of reporting multiple performance metrics for comparability and transparency. For the reader, it is important to use common performance metrics based on the task as a baseline to make it comparable to other techniques in the field. This phenomenon has been observed in most of the papers we have covered. However, some have only reported a single performance metric, such as the AP3 [60] or DeepDetect [13] papers. The other important aspect of performance reporting is the data on which the authors base their performance. Quite a few papers report their performance only on their own data or choose validation datasets from other studies. The second approach is in itself a good measure of performance, but there is still a bias if the authors do not use the datasets of the studies they are comparing their own approach to report their performance. At that point, it is not clear to other researchers whether the datasets from other studies were handpicked because their techniques performed exceptionally well on those datasets or not. To ensure an unbiased evaluation, the proposed technique should be benchmarked using the original datasets employed in the corresponding baseline studies. Of course, this is only possible when comparing techniques with the same input features. We have also observed papers like the KDEAN [65] paper where the reported performance on multiple metrics was only improvements on the order of 1% to 2%. This raises the question of whether the gain is practically meaningful in real experimental settings, especially in the absence of statistical significance testing. Notably, across all the tools and methodologies evaluated in our scoping review, we could not identify a study that employed rigorous statistical tests to validate their reported performance gains.

To address this widespread critical gap, our fifth guideline emphasizes the absolute necessity of statistical testing and comprehensive evaluation metrics. When minor performance gains are reported, it is imperative to apply context appropriate statistical tests, such as the DeLong test for single independent sets or the Wilcoxon signed-rank test for resampled data, to confirm that these improvements are genuine rather than artifacts of random data splitting. Furthermore, this guideline advocates for evaluating model stability through variance testing and explicitly addressing the highly imbalanced nature of proteomics datasets by prioritizing threshold-independent metrics like the AUC PR. Beyond mere discriminative accuracy, we strongly recommend that future studies assess model calibration and perform zero shot testing across independent datasets. Addressing these factors ensures that newly developed AI tools not only are statistically sound but also reliably generalize to new experimental environments, such as different mass spectrometers or biological species.

Regarding the sixth guideline, the description of hyperparameters is very important. These can influence the performance and behavior of the model and are needed to understand and reproduce it. In the tools we collected, hyperparameters were rarely discussed. In the field of computational proteomics, the optimization and understanding of hyperparameters is particularly important due to the diversity of datasets involved.

As stated in the seventh guideline, providing detailed metadata on the data used in the study is of great importance. The factors of utilized search engines, reference database, and data acquisition collectively shape the characteristics of the data and can impact model performance and generalizability. Including this metadata not only enables other researchers to better interpret results but also facilitates more meaningful comparisons across studies and supports reproducibility within the community.

In line with the eighth guideline, mitigating data leakage requires moving beyond classical dataset randomization to address both biological and technical overlaps. If a dataset is partitioned at the peptide level, fragments originating from the exact same parent protein may be distributed across both training and test sets. Because peptides from the same protein share biochemical properties and coexpression patterns, a model can easily memorize these specific features rather than learning generalized biological rules. Therefore, dataset splitting must occur at the protein level, ensuring all peptides from a given protein are assigned exclusively to either the training or the testing partition. However, standard protein level splitting is insufficient when dealing with evolutionary cousins. Proteins in the training and test sets might still share extensive sequence identity. Performing homology clustering before splitting is therefore essential. Tools such as *MMseqs2* can group sequences by evolutionary similarity, allowing entire clusters to be assigned to a single partition based on a strict maximum similarity threshold. This is especially critical when processing multiorganism data to manage shared proteotypic peptides across orthologs. An AA sequence that uniquely identifies a specific protein in one organism might be completely identical to a peptide in an orthologous protein of another species. If these orthologs are split across the training and test sets, the exact peptide sequence bridges the 2 partitions.

Beyond sequence overlap, developers must also address technical confounders, specifically instrument batch leakage and temporal leakage. Biological data are influenced by the specific mass spectrometer and the laboratory environment. If training and test sets contain data generated from the same instrument batch, the model may learn to recognize the unique calibration errors or background noise of that specific run rather than the underlying biology. A truly robust model must be validated on a completely independent batch. Similarly, addressing temporal leakage requires careful consideration of how rapid engineering advancements impact data quality. Randomly shuffling data from older and newer instruments fails to simulate real-world deployment scenarios. While temporal splitting, where a model is trained on historical data and tested on newer instrument generations, can demonstrate forward generalizability, this approach must account for evolving detection limits. The distinction between older and newer instruments is not merely a matter of differing noise profiles or historical artifacts. Modern mass spectrometers achieve higher sensitivity, resolution, and proteomic depth, yielding a fundamentally richer data representation. Consequently, the ground truth itself evolves over time. A peptide that was confidently labeled as undetectable 10 years ago due to hardware limitations might be routinely detectable on a platform today. Researchers must recognize that older datasets may contain false negatives driven by historical instrument constraints rather than true physical properties. When designing temporal holdouts, developers must balance the need to evaluate technological robustness with the reality that newer datasets inherently provide a more accurate and comprehensive biological ground truth. Systematically addressing these complex sources of biological and technical leakage will significantly enhance the credibility and reliability of reported performance metrics in future peptide detectability models.

We encourage researchers to consider and follow these guidelines when designing experiments and developing classifiers for the prediction of detectable and undetectable peptides. We also hope that these recommendations will prove valuable beyond peptide detectability, contributing to improved standards across other areas of computational proteomics.

## Future Research and Development

Future research in this area is expected to focus on 5 major components: method development, data generation and curation, application-driven evaluation, the integration of explainable artificial intelligence (XAI), and the creation of community-curated benchmarks. With respect to method development, the incorporation of peptide structural information into detectability prediction models represents a promising direction, particularly in light of recent advances in computational structural biology. State-of-the-art structure prediction frameworks such as AlphaFold [87], ESMFold [88], and OpenFold [89] now enable large-scale, high-accuracy inference of peptide and protein structures directly from sequence. Structural features derived from such models may provide complementary information to sequence-based representations and improve model generalization across different experimental conditions. While proteins are digested in a standard mass spectrometry workflow, the native 3-dimensional conformation of the parent protein still influences detectability. Specifically, integrating structural predictions allows models to account for predigestion folding effects such as solvent accessibility and intrinsic order. A cleavage site buried deeply within a folded protein core may exhibit significant steric hindrance, rendering it inaccessible to enzymes for digestion such as trypsin. Consequently, a theoretical peptide might be predicted as highly detectable based solely on its sequence, whereas adding structural context would correctly identify it as a missed cleavage product unlikely to be generated.

Furthermore, the 3-dimensional structure of the cleaved peptide itself can provide mechanistic insights for downstream modeling. Longer-chain peptides can retain or adopt secondary structures in solution, which can affect their chromatographic retention time, ionization efficiency, and therefore overall detectability. Representing these 3-dimensional peptide structures as molecular graphs naturally enables the application of graph neural networks. These DL architectures excel at capturing spatially aware physicochemical properties, mapping the geometric arrangement of charge, and hydrophobicity across the potentially folded peptide [90]. By incorporating these spatial and digestion-based structural features, future detectability models can bridge the gap between theoretical sequence properties and actual sample preparation dynamics, thereby improving model generalization across different experimental conditions. Regarding data, future studies should increasingly adopt DIA as a baseline. Modern DIA search engines such as DIA-NN and Spectronaut have achieved high accuracy, robust FDR control, and benefit from well-curated reference proteomes for model organisms such as human and mouse. This enables a more realistic representation of peptide detectability and helps mitigate the inherent protein abundance bias of DDA. However, the influence of PTMs on peptide detectability remains insufficiently characterized. Future work should therefore consider the development of dedicated predictors trained on multi-PTM peptide datasets to systematically assess their impact on detectability. Moreover, well-curated, multiorganism benchmark datasets will be essential for advancing the field. While the total volume of available mass spectrometry data continues to grow, the availability of consistently annotated, high-quality datasets in public repositories such as the ProteomeXchange Consortium remains a limiting factor [85].

To resolve this issue, we propose establishing a structured, community-wide benchmarking dataset. Such a dataset must be intentionally constructed to capture true biological and technical variance by aggregating high-quality empirical data across multiple organisms, diverse mass spectrometry instruments, and distinct acquisition protocols. Specifically, this benchmark should incorporate extensively studied model organisms such as *H. sapiens*, *Saccharomyces cerevisiae*, *M. musculus*, and *E. coli*, as these provide a rich diversity of available datasets across different platforms. While historical DDA data has been instrumental in training the first generation of detectability models and remains a cornerstone of proteomics research, the integration of these historical workflows with modern DIA approaches requires critical reevaluation for benchmarking purposes. For the specific task of training peptide detectability models, DIA offers distinct advantages. DDA relies on stochastic precursor selection, which can bias training data by occasionally missing lower abundance peptides. Consequently, a peptide labeled as undetectable in a DDA dataset might simply represent a missed sampling event rather than true physical undetectability. If a highly accurate predictor is trained on comprehensive DIA data, which captures a greater proteomics depth, and is subsequently benchmarked against DDA data, a profound evaluation conflict arises. The model may correctly classify a peptide as detectable based on its learned rules, but it will be penalized if that peptide was stochastically missed in the ground truth.

Given the challenges of reconciling stochastic sampling with comprehensive fragmentation, we suggest that the field should progressively transition toward utilizing DIA datasets as the primary foundation for the training and validation of future detectability models. While DDA datasets are abundant and historically significant, the reduced false negative rate in DIA provides a cleaner ground truth that is more optimally suited for robust ML. Furthermore, the construction of this benchmark must be paired with a rigorous, stratified evaluation protocol. While a leave-one-species-out evaluation strategy can be used to measure zero shot transferability, predicting peptide detectability is an inherently difficult task that requires large amounts of training data. Completely isolating species during training may deprive the model of biological context. For instance, posttranslational modifications can influence the detectability of a peptide, and because different species exhibit distinct modification patterns, incorporating a mixture of species within the training set allows the model to implicitly learn these complex biological variances. Therefore, we argue that the evaluation framework should instead prioritize strict protein-level and instrument-level holdouts. By ensuring that peptides originating from the same parent protein are never shared between training and validation sets, developers can successfully prevent sequence-based data leakage. Combining this strict protein-wise separation with instrument-level holdouts provides a highly effective and biologically sound mechanism to finally resolve the problem of performance comparisons among competing models.

From an application perspective, future methodological studies should place greater emphasis on demonstrating real-world utility. In addition to reporting model design and performance metrics, studies should critically evaluate the practical benefits of peptide detectability predictions in downstream proteomics workflows. An aspect that remains underrepresented in current research is the development of explainable and interpretable models. Users of detectability prediction tools are concerned not only with predictive accuracy and robustness but also with understanding the underlying reasons for a model’s decisions. Interpretability may reveal structural motifs, physicochemical properties, and AA compositions that govern peptide detectability in mass spectrometry. Previous approaches combining physicochemical descriptors with tree-based models have demonstrated the value of interpretability, and future developments should strive to balance predictive performance with XAI. Another impactful intervention for the field is the establishment of community-curated benchmarks, modeled after successful initiatives in related domains. A prominent example is the Critical Assessment of Structure Prediction (CASP), a community experiment designed to advance methods for computing 3-dimensional protein structures from AA sequences [91]. A similar challenge for peptide detectability would enable fair, head-to-head comparisons between tools and incentivize innovation by shifting the validation burden to an independent entity. In the CASP framework, progress is driven by rigorous blind testing. Organizers identify targets for which experimental structures are resolved but not yet public, requiring participants to submit predictions for independent assessment. Implementing a similar “blind” format for peptide detectability presents unique challenges. Unlike protein folding, which targets a (more or less) deterministic physical structure, peptide detectability is influenced by stochastic sampling, instrumental, and experimental variance. Furthermore, the vast amount of publicly available mass spectrometry data increases the risk of data leakage during model training. To address this, a community challenge should prioritize the use of diverse, newly acquired datasets from multiple laboratories and instrument platforms. This would ensure that models are evaluated on their ability to generalize across different experimental conditions rather than simply recalling sequences from previous training sets. Furthermore, CASP categorizes targets by difficulty into 4 classes, such as free modeling for proteins with no detectable homology to known structures, which is the most difficult class. A peptide detectability challenge could similarly categorize targets by sequence complexity or species rarity to better evaluate model robustness. This direction aligns with the goals of ProteoBench, a centralized, open-source platform designed to bring together developers and users to provide an evolving comparison of state-of-the-art proteomics data processing tools [92]. By enabling the community to develop dedicated benchmarks for specific tasks, ProteoBench increases transparency and reproducibility. Establishing a similar independent validation entity for peptide detectability would allow developers to test newly developed tools privately before adding them as public references, ultimately shifting the burden of proof away from tool developers and toward a transparent, community-led leaderboard. Finally, future studies should more carefully account for the temporal and instrumental context of the training data. Over the past 2 decades, advances in mass analyzer design have led to orders-of-magnitude improvements in detection sensitivity, signal-to-noise ratio, and limits of detection across quadrupole, ion trap, time-of-flight, and Orbitrap mass spectrometers, which have substantially impacted the quality of data used in ML algorithms [93]. Consideration of such technological evolution will be essential for developing robust, transferable detectability models.

## Conclusion

The findings of our comprehensive analysis of 25 AI techniques for peptide detectability indicate that notable progress has been made with respect to accessibility, source code availability, and reproducibility. Nevertheless, there are still significant areas for improvement, particularly with regard to the selection and characterization of datasets, as well as interstudy comparability, due to the use of diverse datasets and performance metrics. In order to address these challenges, we propose the establishment of a standardized, gold standard dataset alongside unified performance metrics, with a view to enhancing cross-study comparisons. Moreover, our recommendations for ML in the field of peptide detectability underscore the significance of data transparency and accessibility, rigorous cross-validation protocols, comprehensive performance reporting, detailed documentation of hyperparameters, detailed metadata, and analysis of potential data leakage. Looking forward, the next generation of peptide detectability models must transcend sequence-based predictions by integrating structural biology insights from frameworks like AlphaFold and embracing DIA and multi-PTM datasets to reduce abundance bias. Furthermore, a shift toward XAI and real-world utility will be essential to ensure that models provide biological interpretability and remain transferable across evolving mass spectrometry platforms. By adhering to these technical standards and pursuing these innovative research directions, the field can achieve enhanced reproducibility and scientific impact, ultimately ensuring the integrity and utility of AI-driven discoveries in proteomics.

## Data Availability

The data used for the study and the figures and the code to create the figures are accessible at https://github.com/SchillingV/Adoption-of-AI-Standards-for-Improved-Peptide-Detectability-in-Computational-Proteomics
