# Supplementary material for: The State of Peptide Detectability in Computational Proteomics and Guidelines for AI Applications
Source: Comput Struct Biotechnol J. 2026 Apr 7;35(1):0037. doi: 10.34133/csbj.0037 (PMC13082577; doi:10.34133/csbj.0037)
Supplement: Supplementary 1 — Table S1 [file csbj.0037.f1.pdf]

Table S1 Overview of peptide detectability tools and their last known update status. The tools are ordered by their original publication year. “Unknown” has been put if the tools were not available anymore or last update notes could not be found.

Year	Technique/Paper	Link	Last Updated	License
2006	[9]	NA	Unknown	NA
2007	[55]	NA	Unknown	NA
2007	PeptideSieve [58]	NA	Unknown	NA
2008	The APEX [11]	https://sourceforge.net/projects/apexqpt/	13.04.2014	open-source
2009	ESPPredictor [59]	https://github.com/genepattern/ESPPredictor?tab=readme-ov-file	30.04.2020	open-source
2010	STEPP [57]	NA	Unknown	NA
2010	IL2 [52]	NA	Unknown	NA
2011	CONSeQuence [10]	NA	Unknown	NA
2014	PeptideRank [53]	NA	Unknown	NA
2015	PPA [63]	NA	Unknown	NA
2015	PREGO [33]	https://github.com/briansearle/intensity_predictor?tab=readme-ov-file	11.01.2022	open-source
2017	[49]	NA	Unknown	NA
2018	d::pPop [48]	NA	Unknown	NA
2019	AP3 [60]	http://fugroup.amss.ac.cn/software/AP3/AP3.html	01.05.2019	unspecified
2020	DeepMSPeptide [50]	https://github.com/vsegurar/DeepMSPeptide?tab=MIT-1-ov-file	09.08.2019	open-source
2020	DeepDIA [64]	https://github.com/lmsac/DeepDIA/?tab=readme-ov-file	23.10.2022	open-source
2021	PepFormer [8]	https://github.com/WLYLab/PepFormer	15.03.2021	open-source
2021	CapsNet [54]	https://github.com/yuminzhe/yuminzhe-Prediction-of-peptide-detectability-based-on-CapsNet-and-CBAM-module	21.12.2021	NA
2022	PD-BertEDL [61]	NA	Unknown	NA
2023	DeepDetect [13]	http://fugroup.amss.ac.cn/software/DeepDetect/DeepDetect.html	27.02.2023	non-commercial use
2023	DbyDeep [65]	https://github.com/HanyangBISLab/DbyDeep	08.04.2023	open-source
2023	PeptideRanger [56]	https://github.com/rr-2/PeptideRanger?tab=readme-ov-file	17.05.2023	open-source
2024	KDEAN [66]	https://github.com/Xiaocai-Zhang/KDEAN	16.11.2023	NA
2024	DeepPD [67]	https://github.com/leonern/DeepPD	15.09.2023	open-source
2025	Pfly [68]	https://github.com/wilhelm-lab/dlomix/tree/main/pretrained_models	28.04.2025	open-source
